# Biomechanical Analysis of the Effects of Bilateral Hinged Knee Bracing

**DOI:** 10.3389/fbioe.2016.00050

**Published:** 2016-06-16

**Authors:** Hangil Lee, Dokyeong Ha, Yeoun-Seung Kang, Hyung-Soon Park

**Affiliations:** ^1^Mechanical Engineering Department, Korea Advanced Institute of Science and Technology, Daejeon, Korea

**Keywords:** knee brace, biomechanics, sports injury, motion capture, force plate, kinetics

## Abstract

This research analyzed the effect of bilateral hinged knee braces on a healthy knee from a biomechanical frame *in vivo*. This was accomplished by fitting a knee brace with two customized wireless force/torque (*F*/*T*) sensors that could readily record force and torque during live motion, while the kinetics at the knee were computed using the inverse dynamics of the motion capture and force plate data. Four tasks to test the brace’s effects were drop vertical jumping, pivoting, stop vertical jumping, and cutting. The results showed that the hinges in the knee brace can absorb up to 18% of the force and 2.7% of the torque at the knee during various athletic motions. Thus, the hinges demonstrated minimal effect in reducing the mechanical load on the knee. There were limitations concerning the consistency of the motions performed by the subjects during the trials and the influence of the other portions of the brace to evaluate the overall effectiveness of the brace as a whole. Future works may incorporate a fatigue protocol and injured subjects to better determine the effects of the brace. There is still a need for more research on the biomechanical influence of knee braces to develop safer and more effective products.

## Introduction

The knee is an essential joint for everyday motions. It is the largest joint in the body, and one of the most easily injured. It is made up of four main parts: bones, cartilage, ligaments, and tendons. Significant ligament injuries or arthritis can cause severe discomfort for many people. In particular, athletes are extremely susceptible to knee injuries; many continually fall victim to critical knee ligament tears every year. Anterior cruciate ligament (ACL) and medial collateral ligament (MCL) injuries are infamous for abruptly ending the careers of many devoted athletes. In the USA alone, there are over 100,000 cases of ACL injuries annually, with a majority of those occurring from playing sports (Mall et al., 2014). To improve the safety and livelihood of athletes, countless studies have investigated preventative measures to protect the knee. One of the most controversial issues for knee protection is the use of a prophylactic brace. This type of knee brace is designed to protect healthy athletes against knee injuries, as opposed to functional braces that aid in rehabilitating patients who have already suffered from a serious injury. The most common type of prophylactic knee braces is a bilateral hinged brace that consists of a wrap-around sleeve with two hinges inserted into the sides.

Earlier studies have focused on following different athletes throughout the course of a sports season, but little research has been done to quantitatively analyze the biomechanical force and torque involved in using a prophylactic brace. Many of these studies have shown conflicting results on the effectiveness of prophylactic knee braces (Paluska and McKeag, 2000). As a result, there is no concrete evidence to suggest a link between the usage and reduced injury. However, many sports programs still encourage the use of these costly braces, even though they show limited performance for certain motions. There simply have not been enough studies yet that have provided sufficient evidence to support the use of prophylactic knee braces because of the limitations of testing live athletes.

In addition, knee braces are not only applicable to athletes. Osteoarthritis (OA) in the knee is one of most prevalent forms of arthritis, and it can make simple tasks a burden for the elderly. A recent study found that 16% of the population over the age of 45 years in the USA had symptomatic knee OA (Jordan et al., 2009). To treat this medical symptom, non-invasive treatment methods are becoming popular, and one option is the use of knee braces. Many studies have demonstrated a reduction in pain and improved gait performance due to stabilization from braces, but again, there is a lack of biomechanical analysis evaluating how a brace acts to relieve pain.

Although it is unclear how knee braces distribute force during activity, there has been plenty of research highlighting the types of force that lead to knee ligament injuries. A brief understanding of these causative forces will clarify which force to focus on for injury prevention. An accumulation of force and circumstances lead to knee injury, but the primary force believed to load the ACL in particular is axial compressive force paired with an increased tibial posterior slope (Boden et al., 2010). This leads to an anterior tibial translation that is known to be a significant causative factor in ligament tears. In addition, many knee injuries occur due to dynamic knee valgus when planting. Knee valgus due to OA is also a leading cause of pain and discomfort for patients.

The purpose of this research was to analyze the effect of bilateral hinged knee braces from a biomechanical frame *in vivo* by utilizing a knee brace fitted with two customized force/torque (*F*/*T*) sensors. As mentioned above, it is difficult to generalize the cause of all ligament injuries or discomfort from OA to a single force. Therefore, this study focused on a biomechanical analysis of the changes in force and torque at the knee due to bracing.

## Materials and Methods

Five healthy male subjects [age 24.5 ± 6.5; mean BMI 24.1 (SD 2.4)] with no previous history of knee injury participated in this study. None of the subjects were professional athletes, although all five subjects participated in sports (soccer, basketball, or weightlifting) recreationally about 3 h a week. All experimental protocols were explained beforehand, and each subject signed a consent form approved by the Institutional Review Board at Korea Advanced Institute of Science and Technology (KAIST).

Each subject was given sports performance clothing and asked to complete all the tasks barefoot to eliminate any influence of external variables from shoes. An experienced medical professional attached reflective markers on specific bony landmarks located on the second metatarsal head, lateral malleolus, calcaneus, lateral knee joint line, and pelvis for each leg of the subject.

The subjects were asked to complete four tasks: drop vertical jumping (DVJ), stop vertical jumping (SVJ), pivoting, and cutting. The set of tasks was repeated under unbraced and braced conditions, and each trial was repeated three times. The subjects were allowed to practice each movement until they were comfortable with the motions. The tasks followed the order of unbraced (DVJ, pivoting, SVJ, and cutting) and then braced (DVJ, pivoting, SVJ, and cutting).

**Figure 1:**
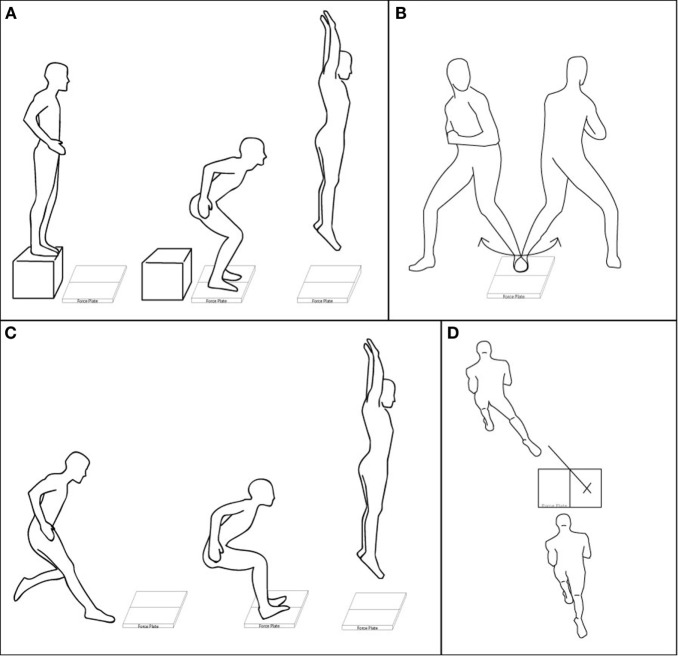
**Illustrations of the four tasks**. **(A)** DVJ: drop off of a box and jump off the force plates, **(B)** pivoting: pivoting off one foot planted on a force plate, **(C)** SVJ: running approach, landing with each foot on a force plate and jumping vertically, and **(D)** cutting: running toward a force plate, planting right foot on the X and pushing off diagonally in the direction of the tape.

The DVJ involved the subject starting on an elevated surface (20 cm), dropping down with each foot on a separate force plate and immediately jumping straight up for maximum height (Figure 1A). This task was shown to exhibit a high within-session reliability (Hewett et al., 2005) and commonly used to reliably measure athletes’ vertical jump. For the SVJ, the subject was instructed to take a running three step approach, leap forward onto the force plates, land on both feet, and then jump straight up (Figure 1C). About 70% of non-contact sports related accidents (ACL injuries) were a result of stop-jump motions (Yu et al., 2004), so these two jumping tasks were included in this study.

Pivoting involved the subject planting their non-dominant foot on a force plate and pivoting around it with their dominant leg (Figure 1B). The subjects were asked to keep one foot firmly planted without slipping while rotating their hips in order to plant their foot at the furthest possible point of rotation in both directions to the beat of a metronome set at 50 bpm. While the subjects were practicing this movement, a small piece of tape was placed on the ground marking the maximum foot positions, so the subjects could aim for a reference point during the actual task. The data were collected in the middle of iterations for the most natural series of pivots.

The last cutting task was based on a previous study (Besier et al., 2001). A line of tape angled 45° off the centerline of a single force plate was used as a reference line for the cutting direction. The subjects were instructed to take a three-step running approach jump onto the plate and land on their dominant foot (right for all subjects), while using that same leg to push off into the designated tape direction (Figure 1D). Both pivoting and cutting tasks were included for their tendency to induce internal tibial rotation and valgus movements.

To capture the motion data, eight Vicon Vantage cameras (Vicon Motion Systems Ltd., UK) were set up around two AMTI AccuGait force plates (Advanced Mechanical Technology Inc., MA, USA). Additional tiles were placed around them to create a level surface for the tasks. The force plates recorded ground reaction force (GRF), torque, and center of pressure data through the Vicon Nexus software at 1 kHz, while the Vicon cameras captured marker position at 100 Hz.

**Figure 2:**
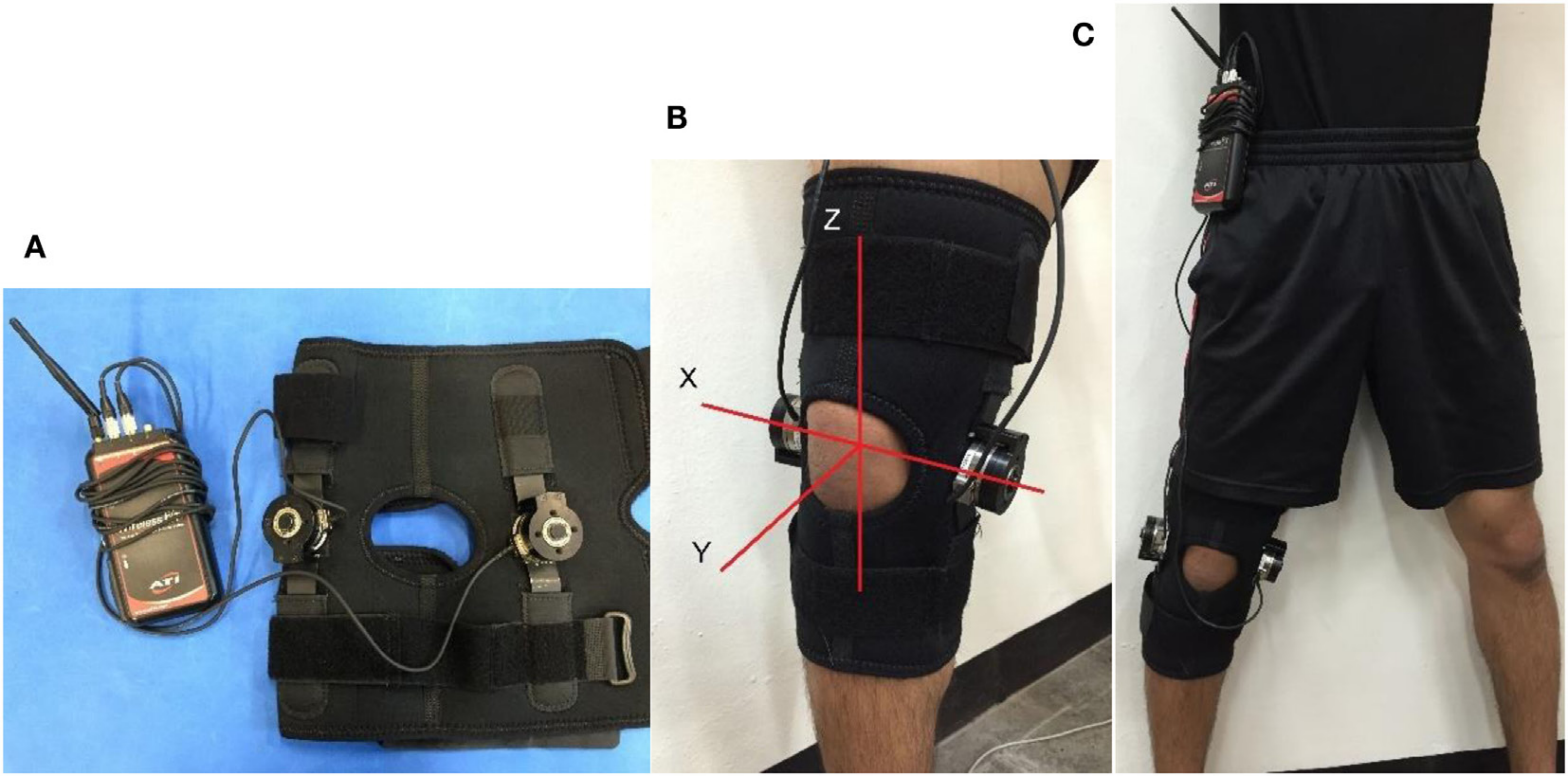
**Customized *F*/*T* sensor hinge: (A) the bilateral hinges with *F*/*T* transmitter, (B) fitted knee brace with assigned coordinate system, and (C) braced condition with wireless transmitter clipped onto waist**.

For the braced condition, a prophylactic knee brace with two *F*/*T* sensors was used. To measure the external force and torque absorbed by the brace’s hinges, two *F*/*T* sensors (Mini45, ATI Industrial Automation, NC, USA) were adjusted to fit into the inner and outer sleeves of the Hely Weber Velocity Hinged Knee Brace (Weber Orthopedic Inc., DBA Hely & Weber, CA, USA) (Figure 2A). The original brace consisted of a sleeve and two hinges. Each subject was given an appropriately sized sleeve (all medium or large), and the sleeve was applied to the knee tightly. The size of the original hinges was the same for all sleeve sizes, so the same *F*/*T* sensors were used for all subjects (Figure 2B).

These sensors were connected to a wireless transmitter that could transmit raw data over WiFi to a laptop program. They were able to rotate freely along the lateral knee joint line and did not hinder knee flexion. The transmitter was clipped onto the waistline and provided little bulk (Figure 2C). A cluster of markers placed on the outer sensor replaced the corresponding knee joint marker, and the thickness of the sensor (32 mm) was accounted for in data analysis.

**Figure 3:**
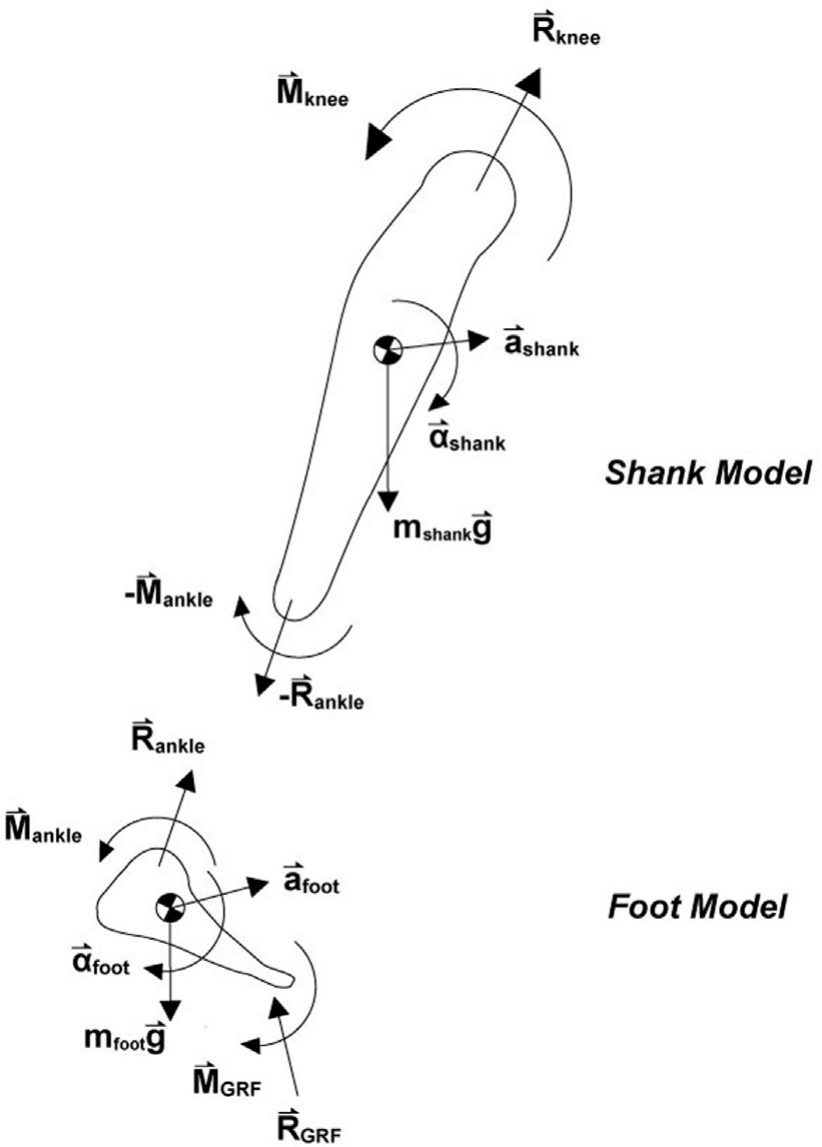
**To calculate the kinetics at the knee, the lower extremity was modeled into a foot and shank segment based on marker data**. Inverse dynamics was used with the ground reaction force and torque to compute the resulting force and torque in the knee along each of the three axes of the coordinate system described above.

The *F*/*T* sensor data collection program was run simultaneously using the Vicon Nexus program for synchronous data collection. The force plate and motion data were used to compute the kinetic variables at the knee using inverse dynamics, while the *F*/*T* sensor data directly transmitted force and torque measurements that were calibrated to newton and newton meter, respectively. The lower extremity was modeled into foot and shank segments, as described in Figure 3. The dynamics of the foot segment may be described using the following equations of motion:

$$\begin{array}{c} {\vec{R}}_{\mathrm{GRF}}+m_{\mathrm{foot}}\vec{g}+{\vec{R}}_{\mathrm{ankle}}=m_{\mathrm{foot}}{\vec{a}}_{\mathrm{foot}},\\ {\vec{M}}_{\mathrm{GRF}}+{\vec{M}}_{\mathrm{ankle}}=I_{\mathrm{foot}}{\vec{\alpha }}_{\mathrm{foot}}. \end{array}$$

Since the GRF and torque from the force plates $\left({\vec{R}}_{\mathrm{GRF}},{\vec{M}}_{\mathrm{GRF}}\right)$ as well as the mass, inertia, and accelerations $\left(m_{\mathrm{foot}},I_{\mathrm{foot}},{\vec{\alpha }}_{\mathrm{foot}}\right)$ are known, the force and torque at the ankle $\left({\vec{R}}_{\mathrm{ankle}},{\vec{M}}_{\mathrm{ankle}}\right)$ can be found. Due to Newton’s third law of action and reaction forces, the reaction force and torque at the ankle in the shank model are equal and opposite to those of the foot model. Thus, the shank model can be written as:

$$\begin{array}{c} {\vec{R}}_{\mathrm{knee}}+m_{\mathrm{shank}}\vec{g}-{\vec{R}}_{\mathrm{ankle}}=m_{\mathrm{shank}}{\vec{a}}_{\mathrm{shank}},\\ \text{​}{\vec{M}}_{\mathrm{knee}}-{\vec{M}}_{\mathrm{ankle}}=I_{\mathrm{shank}}{\vec{\alpha }}_{\mathrm{shank}}. \end{array}$$

This can be solved for the force and torque at the knee by rewriting:

$$\begin{array}{l} {\vec{R}}_{k\mathrm{nee}}=m_{\mathrm{shank}}{\vec{a}}_{\mathrm{shank}}-m_{\mathrm{shank}}\vec{g}+{\vec{R}}_{\mathrm{ankle}},\\ {\vec{M}}_{\mathrm{knee}}=I_{\mathrm{shank}}{\vec{\alpha }}_{\mathrm{shank}}+{\vec{M}}_{\mathrm{ankle}}. \end{array}$$

In this manner, the kinetics of the knee were computed using an inverse dynamics code written in MATLAB that utilized the motion data from the Vicon cameras as well as the force, torque, and center of pressure measurements obtained from the force plate.

For the braced trials, the knee marker offset by the thickness of the *F*/*T* sensor (32 mm) in the brace was accounted for by placing a cluster of three markers on the hinge point and computing the cross product of two segments formed from the cluster. This produced a vector pointing inward along the axis of the offset, and the thickness of the sensor was subtracted from the position along the vector. In calculating the inertia tensor matrices, scaling factor values for the inertial parameters of body segments were adopted from a literature (Dumas et al., 2007).

For describing knee joint kinetics, the *Z* and *Y* axes were aligned with the tibia and the flexion axis, respectively (Figure 2B), which was akin to the coordinate system attached to the tibia in the joint coordinate system (Grood and Suntay, 1983). Since the coordinate system of the *F*/*T* sensor exactly matched the knee joint coordinate system, no transformation for the *F*/*T* data was needed. The biomechanical model used for the force and torque at the orthosis was “With Brace condition” (human knee model + orthosis model) − “without brace condition” (human knee model only).

## Results

**Figure 4:**
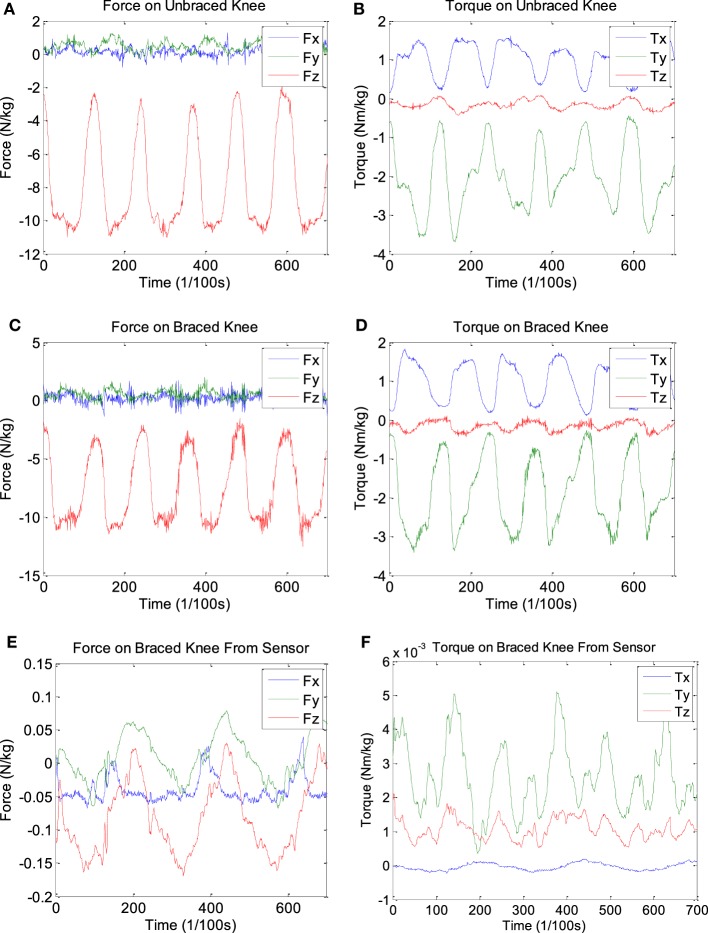
**Representative pivoting data**. Three sets of graphs for the **(A)** force and **(B)** torque of the braced condition, **(C)** force and **(D)** torque of the unbraced condition, and **(E)** force and **(F)** torque for the *F*/*T* sensor data.

Representative force and torque results obtained under “With Brace condition” and “without brace condition,” and from the *F*/*T* sensor in the pivoting task are shown in Figure 4. The force results were all normalized to newton per kilogram, and the torque results to newton meter per kilogram.

The peak-to-peak values of force and torque in the *x*, *y*, and *z* direction were measured for every trial, averaged, and normalized with the body weight of each subject. The SDs between the averages of each subject were also computed. The results are summarized in Table 1. The force/torque at the knee joint under the “W/O Brace condition” can be obtained directly from the inverse dynamics solution. The force/torque under the “With Brace condition” was obtained by subtracting the force/torque measured by the *F*/*T* sensor from the inverse dynamics solution since the inverse dynamics calculated the force/torque taken by both knee joint and the hinge. The maximum percentage of force and torque the sensor absorbed was also calculated for the “% *F*/*T* taken by Hinge” column:

%F/T  taken by Hinge=F/TsensorWith Brace×100.

**Table 1:** **Averaged and normalized peak-to-peak force (newton per kilogram) and torque (newton meter per kilogram) values along with the SDs at the knee joint for the W/O Brace condition and With Brace condition**.

Task/component	*F*/*T* at knee joint (W/O Brace condition)	*F*/*T* at knee joint (With Brace condition)	% *F*/*T* taken by hinge
Normalized Fx (DVJ, N/kg)	5.84 (SD ± 2.59)	6.04 (SD ± 1.71)	3.74
Normalized Fy (DVJ, N/kg)	3.09 (SD ± 0.63)	2.32 (SD ± 0.52)	10.17
Normalized Fz (DVJ, Nm/kg)	19.06 (SD ± 4.82)	17.77 (SD ± 3.72)	1.52
Normalized Tx (DVJ, Nm/kg)	4.97 (SD ± 1.41)	4.98 (SD ± 1.47)	0.02
Normalized Ty (DVJ, Nm/kg)	4.33 (SD ± 1.76)	4.27 (SD ± 0.77)	0.34
Normalized Tz (DVJ, Nm/kg)	1.10 (SD ± 0.65)	0.66 (SD ± 0.08)	0.89
Normalized Fx (Pivot, N/kg)	1.63 (SD ± 0.92)	1.52 (SD ± 0.48)	5.48
Normalized Fy (Pivot, N/kg)	1.11 (SD ± 0.40)	0.88 (SD ± 0.35)	18.19
Normalized Fz (Pivot, Nm/kg)	9.72 (SD ± 1.57)	9.29 (SD ± 1.38)	2.20
Normalized Tx (Pivot, Nm/kg)	1.25 (SD ± 0.66)	2.45 (SD ± 0.93)	0.02
Normalized Ty (Pivot, Nm/kg)	2.12 (SD ± 0.99)	2.90 (SD ± 0.69)	0.19
Normalized Tz (Pivot, Nm/kg)	0.34 (SD ± 0.09)	0.45 (SD ± 0.09)	0.55
Normalized Fx (SVJ, N/kg)	8.85 (SD ± 2.92)	8.46 (SD ± 1.62)	2.93
Normalized Fy (SVJ, N/kg)	2.53 (SD ± 0.72)	2.68 (SD ± 0.82)	8.17
Normalized Fz (SVJ, Nm/kg)	17.89 (SD ± 2.85)	17.92 (SD ± 3.18)	1.63
Normalized Tx (SVJ, Nm/kg)	4.51 (SD ± 1.54)	5.49 (SD ± 1.96)	0.02
Normalized Ty (SVJ, Nm/kg)	4.32 (SD ± 0.74)	6.01 (SD ± 1.27)	0.23
Normalized Tz (SVJ, Nm/kg)	1.17 (SD ± 0.68)	1.86 (SD ± 0.94)	0.26
Normalized Fx (Cut, N/kg)	7.04 (SD ± 1.79)	8.32 (SD ± 2.97)	2.39
Normalized Fy (Cut, N/kg)	6.60 (SD ± 1.35)	6.07 (SD ± 0.88)	3.47
Normalized Fz (Cut, Nm/kg)	19.52 (SD ± 3.67)	18.94 (SD ± 2.52)	1.47
Normalized Tx (Cut, Nm/kg)	4.24 (SD ± 2.21)	6.30 (SD ± 2.01)	0.02
Normalized Ty (Cut, Nm/kg)	5.08 (SD ± 1.37)	6.29 (SD ± 0.95)	0.22
Normalized Tz (Cut, Nm/kg)	1.07 (SD ± 0.38)	1.86 (SD ± 0.86)	2.75

*“% F/T taken by Hinge” refers to the maximum percentage of force and torque the hinge absorbed in comparison to the force/torque at knee joint for the With Brace condition*.

It should be noted that the *F*/*T* sensor was hinged to rotate in the *y* − *z* plane, so *x* torque recorded was negligible.

As seen in Table 1, the knee brace absorbed a small fraction of the total force and torque present at the knee joint. The force and torque values obtained under the unbraced and braced conditions were similar in most cases, and the highest force for all tasks was in the *z* direction, as expected.

## Discussion and Conclusion

The customized *F*/*T* sensors allowed for direct measurement of the force and torque absorbed by the hinges in the bilateral hinged knee braces. The wireless receiver enabled portable application of a sensor during live motions and provided minimal bulk to the subject. The measurements of the sensor were calibrated before the experiments were conducted, and they were confirmed again afterward to ensure the results would be in the appropriate units.

A comparison of the results of the unbraced, braced, and sensor data shows that the brace hinge plays a minor role in reducing the force and torque experienced in the knee during athletic motions. The amounts of force absorbed ranged from 1 to 18%, while the amounts of torque absorbed were even smaller, ranging from 0.01 to 2.7%. The *x* torques absorbed were especially low because the hinges were designed to allow free movement in that plane of motion. Although this hinge had a locking mechanism like most over-the-shelf braces, the tasks did not require extreme knee flexion that would force locking.

In many cases, the force or torque was greater without the device. The reason this occurred in spite of wearing the knee brace is that each trial may have been subjected to varying intensities. The subjects were trained for each task to reduce the discrepancy between trials, but it was impossible for each motion to be done at the exact same intensity *in vivo*. Between the two trial conditions, the general trend in force and torque values was the same and the minor effects of the brace were observable in the *F*/*T* percentage results.

In our experiment, there were two sources of estimation error. First, we assumed that the subjects made consistent movements over the multiple trials. For example, in the DVJ and SVJ tasks, the force and torque values obtained at the knee joint would vary if the subjects jumped from different heights. We checked the variation in jumping height for each trial and found that the variation was within 1% for both unbraced and braced conditions for the DVJ test.

For the SVJ test, the unbraced and braced conditions showed variation within 3 and 2%, respectively. Similarly, for the cutting motion, the variation in approaching speed was within 7% for the unbraced condition and 4% for the braced condition. In the pivoting task, we used a metronome to help the subjects make the pivoting motion with the same rhythm. The variation in the pivoting period was <1% for both unbraced and braced conditions, which was checked by the force/torque data obtained from the force plate.

Another source of estimation error was sensor noise and/or resolution. For motion data collected by the Vicon motion capture system, the position resolution was 0.24 mm, which was about 0.05% of the movements made in the four tasks under both braced and unbraced conditions. The force plate resolution was set to 0.01 N for force and 0.0001 Nm for torque. These were <0.002% of the force and torque values measured for the four tasks under both conditions. The *F*/*T* sensor was used only for the braced condition, and the force resolution of the *F*/*T* sensor was 0.25 N for force and 0.005 Nm for torque. This was corresponded to 1.4 and 0.52% of the force and torque values measured for the four tasks, respectively. Another error that was hard to quantify was from the sliding of soft tissue under the skin where the reflective markers were placed. Additionally, although the brace was tightly wound around the knee, slippage seemed to cause slight misalignment between the orthosis and the knee joint.

It is important to note that a conclusion on the overall effectiveness of knee bracing cannot be made because this study addressed only one component of the bracing mechanism, namely, the hinges. Earlier research has reported that a neoprene brace sleeve itself provides indirect benefits, including heightened proprioception (Herrington et al., 2005) and improved balance for OA patients (Chuang et al., 2007). Further research is needed to quantitatively measure the effects of this compression on knee stability.

Measuring the pressure distribution inside the sleeve may provide additional data for evaluation, but there is a technical challenge to implement pressure measurements without impeding natural knee motions. There is the possibility of using commercial pressure sensors inside the sleeve, but users may experience hindered movement and discomfort due to the thickness and stiffness of the sensor. Making a soft sleeve with an embedded pressure sensor may help, but such equipment is not currently available.

This paper focused on the quantitative kinetics of knee bracing on healthy subjects to evaluate the effectiveness of braces in injury prevention. One of the difficulties in conducting experiments *in vivo* is that extreme forces at high intensities cannot be observed without endangering the subject. The tasks designed in this paper are known to cause a high incidence of injury in sports; however, they provide little threat to the subject when done at low intensities. Thus, healthy knee ligaments may be able to handle the resulting force and torque without any need to utilize a brace. This outcome seemed to explain why our hinges were shown to have little effect on the overall kinetics of the knee.

A possible method to increase the effect of the brace is to design a fatigue protocol for the subject to complete before each task. A study done by Chappell et al. (2005) showed an increase in injury prone landing posture during stop jumps in a fatigued state. This is reflected in athletes who exhibit sluggish motions when exhausted. Therefore, the healthy subjects may demonstrate more benefits from wearing a knee brace in a fatigued state without exerting extreme levels of force.

Patients who have suffered knee ligament injuries may also experience greater benefits from utilizing a knee brace. Unlike healthy subjects, their injury will cause a greater dependence on the external support of a knee brace, and they may demonstrate greater force and torque at the brace. More research is needed in this area of injured patients to quantitatively evaluate the biomechanical effect of knee bracing.

In conclusion, our wireless *F*/*T* sensor showed that the hinges in knee braces could absorb up to 18% of the force and 2.7% of the torque in knees during various athletic motions. There are limitations in conducting research *in vivo* with regards to the safety of the subjects, and a need for more research to evaluate the effects of other components of knee braces on injury prevention. Future works may involve a fatigue protocol and works focusing on injured patients. This may better highlight the effects of bracing on knee stabilization. The results from this study may be interpreted from an injury prevention viewpoint and provide greater details on those implications. Understanding on the specific biomechanical effects of bracing would allow for personalized design of knee braces that could address each individual’s weaknesses and risk factors. This would lead to the development of more effective and safer braces for both athletes and OA patients.

## Author Contributions

Mr. HL conducted the experiment, analyzed data, and wrote the manuscript. Mr. DH conducted the experimental setup, analyzed data, and revised the manuscript. Dr. Y-SK designed the experimental protocol. Prof. H-SP initiated the idea, designed the study, and supervised all procedures, including experiment, data analysis, and manuscript writing.

## Conflict of Interest Statement

The authors declare that the research was conducted in the absence of any commercial or financial relationships that could be construed as a potential conflict of interest.
